# Clinical and Therapeutic Aspects of Sideroblastic Anaemia with B-Cell Immunodeficiency, Periodic Fever and Developmental Delay (SIFD) Syndrome: a Systematic Review

**DOI:** 10.1007/s10875-022-01343-0

**Published:** 2022-08-19

**Authors:** Ilaria Maccora, Athimalaipet V. Ramanan, Daniel Wiseman, Edoardo Marrani, Maria V. Mastrolia, Gabriele Simonini

**Affiliations:** 1 Rheumatology Unit, Meyer Children’s University Hospital, Viale Pieraccini 24, 50139 Florence, Italy; 2 NeuroFARBA Department, University of Florence, Viale Pieraccini 24, 50139 Florence, Italy; 3 Bristol Royal Hospital for Children and Translational Health Sciences, University of Bristol, Bristol, UK; 4 Department of Haematology, Royal Manchester Children’s Hospital, Manchester, UK

**Keywords:** SIFD, Immunodeficiency, Autoinflammatory disease, Sideroblastic anaemia, Recurrent fever, Etanercept

## Abstract

**Background and Purpose:**

Sideroblastic anaemia with B-cell immunodeficiency, periodic fever and developmental delay (SIFD) syndrome is a novel rare autoinflammatory multisystem disorder. We performed a systematic review of the available clinical and therapeutics aspects of the SIFD syndrome.

**Methods:**

A systematic review according to PRISMA approach, including all articles published before the 30^th^ of July 2021 in Pubmed and EMBASE database, was performed.

**Results:**

The search identified 29 publications describing 58 unique patients. To date, 41 unique mutations have been reported. Onset of disease is very early with a median age of 4 months (range 0–252 months). The most frequent manifestations are haematologic such as microcytic anaemia or sideroblastic anaemia (55/58), recurrent fever (52/58), neurologic abnormalities (48/58), immunologic abnormalities in particular a humoral immunodeficiency (48/58), gastrointestinal signs and symptoms (38/58), eye diseases as cataract and retinitis pigmentosa (27/58), failure to thrive (26/58), mucocutaneous involvement (29/58), sensorineural deafness (19/58) and others. To date, 19 patients (35.85%) died because of disease course (16) and complications of hematopoietic cell stems transplantation (3). The use of anti-TNFα and hematopoietic cell stems transplantation (HCST) is dramatically changing the natural history of this disease.

**Conclusions:**

SIFD syndrome is a novel entity to consider in a child presenting with recurrent fever, anaemia, B-cell immunodeficiency and neurodevelopmental delay. To date, therapeutic guidelines are lacking but anti-TNFα treatment and/or HCST are attractive and might modify the clinical course of this syndrome.

**Supplementary Information:**

The online version contains supplementary material available at 10.1007/s10875-022-01343-0.

## Introduction

Sideroblastic anaemia with B-cell immunodeficiency, periodic fever and developmental delay (SIFD) syndrome is a novel rare multisystem disorder to be considered in infants and children [1, 2]. In 2013 and 2014, Wiseman et al. and Chakraborty et al. reported the first 12 patients with SIFD syndrome with a homozygous or compound heterozygous mutation in TRNT1 gene [1, 2]. TRNT1 encodes for a transfer RNA (tRNA) nucleotidyl-transferase 1 [2] that is responsible for the 3′ end adding trinucleotide sequence consisting of cytosine-cyto-sine-adenine (CCA). It is necessary for mitochondrial and cytosolic protein syntheses in positioning tRNAs on ribosomes and in terminating protein translation. Therefore, this protein has a crucial role in the protein maturation, in the mitochondrial electron transport chain and in the prevention of intracellular oxidative stress. After the first reports, the phenotypic spectrum and the insight in pathophysiology of this syndrome have been widely expanded [3, 4]. The aim of this systematic review is to describe the phenotype spectrum and the detected mutations currently described in literature.

## Materials and Methods

This systematic review was conducted in accordance with the Preferred Reporting Items for Systematic Reviews and Meta-analyses (PRISMA) guidelines. A systematic search was conducted to identify studies reporting cases of patients with SIFD syndrome. The literature search and evaluation were performed in 2 databases, MEDLINE (PubMed) and EMBASE, for articles published up to the 30th of July 2021. The key words, inclusion and exclusion criteria, type of study to include, the methodology used to select papers and extract data were widely described in the Supplementary material.

## Results

**Fig. 1:**
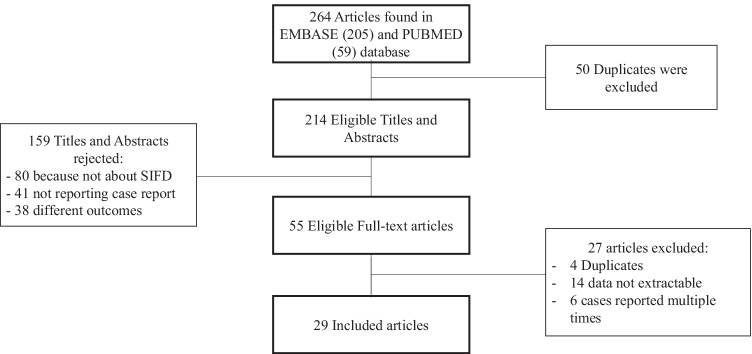
PRISMA flow diagram. Presentation of the procedure of literature searching and selection with numbers of articles at each stage

The search identified 264 eligible articles, of which 50 were duplicates, and, after title and abstract screening, 159 articles were excluded. The full-text length of 55 articles was assessed for eligibility and 29 articles were included (Fig. 1; Supplementary Table 1). The articles 1 and 2 were included because article number 1 describes the phenotypic characteristics of patients reported in article 2 where the genetic results are shown.

### Genetic Characteristic

**Fig. 2:**
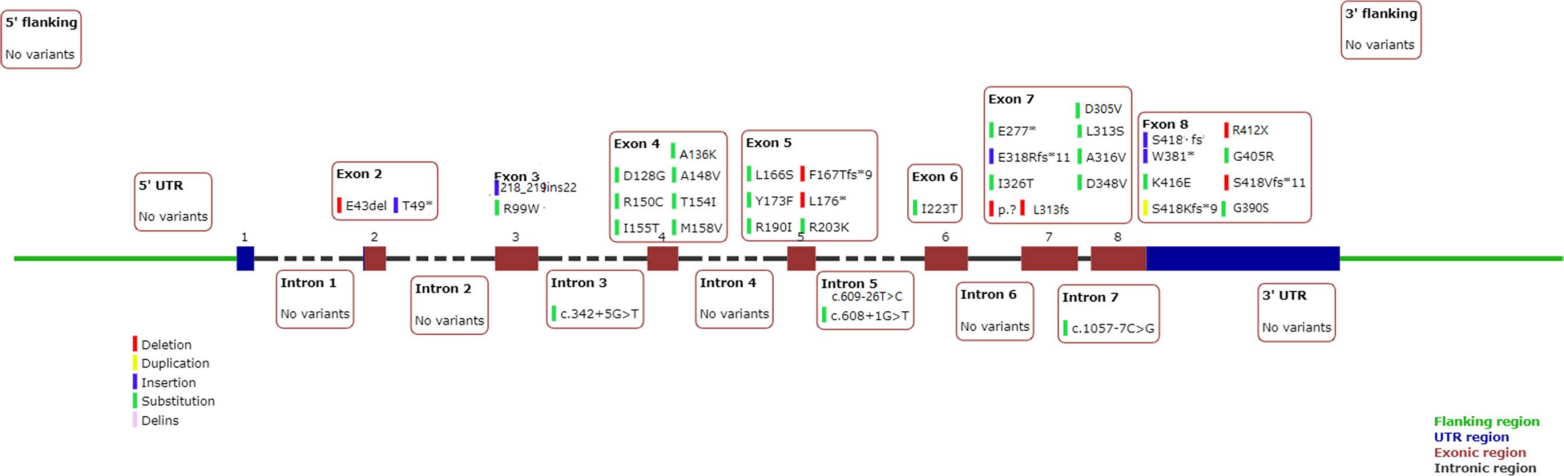
Schematic representation of TRNT1 gene, with the different mutations reported

TRNT1 is composed by 7 introns and 8 exons, most of the mutations reported were found in the exonic regions (Fig. 2). Forty-one unique mutations have been reported to date: 17 patients presented a homozygous mutation in TRNT1, while 38 showed compound heterozygosity and 3 were heterozygous for a single mutation (Tables 1, 2 and 3). The type of reported mutations are more frequently missense mutations. Cases of frameshift and splicing mutations are however identified. These lasts were only in the intronic regions, conversely to the other types reported across the entire gene. When homozygosity was evaluated, the found mutation is typically a missense mutation with the exception of one patient where a homozygosity for frameshift mutation was discovered c.218_219ins22 NA Exon 3 [5].

**Table 1:** Clinical and laboratory description of patients with SIFD. *F*, female; *M*, male; *SA*, sideroblastic anaemia; *m*, months; *y*, years; *IQ*, intelligence quotient

Pts ID and [reference]	Sex	Mutation	Age at onset	Fever	Impaired growth	Hematologic features	Immunologic features	Hypogammaglobulinemia	B-cell (low-normal-high)	Neurologic features with age at onset	Gastrointestinal features	Ophthalmologic	Hearing	Skin and hair involvement	Musculoskeletal	Others
1* [1, 2, 4]	F	Homozygous c.569G.T p.R190I Exon 5 Missense	12 m	+	+	SA	B lymphopenia, Hypogammaglobulinemia, fall T and NK cells	+	Low	Developmental delay. Neuroimaging: cerebral atrophy	During fever episodes Gastrointestinal upset (abdominal pain, diarrhoea and vomiting)	Retinitis pigmentosa	Sensorineural deafness	Ichthyotic skin changes		Nephrocalcinosis Aminoaciduria + hyperalaninemia, Lactic acidosis, electrolytes imbalance
2* [1, 2, 4]	M	Homozygous c.569G.T p.R190I Exon 5 Missense	2 m	+	+	SA	Blymphopenia, Hypogammaglobulinemia, fall T and NK cells	+	Low	Developmental delay. Neuroimaging: cerebral atrophy	During fever episodes Gastrointestinal upset (abdominal pain, diarrhoea and vomiting)	Retinitis pigmentosa	Sensorineural deafness			Nephrocalcinosis, Aminoaciduria + hyperalaninemia, Lactic acidosis electrolytes imbalance
3 [1, 2]	F	Compound heterozygous: c.668 T.C p.I223T Exon 6 Missense c.1057-7C.G NA Intron 7 Splicing	Neonatal	+		SA	Immunodeficiency B-cell	+	Low	Delopmental delay, seizures. Neuroimaging: cerebral atrophy, decreased cerebbalr perfusion	During fever episodes Gastrointestinal upset (abdominal pain, diarrhoea and vomiting)			Brittle hair		Splenomegaly, Renal tubular Fanconi syndrome
4 [1, 2]	M	Compound heterozygous: c.668 T.C p.I223T Missense c.1057-7C.G NA Intron 7 Splicing	3 w	+	+	SA	Immunodeficiency B-cell	+	Low	Developmental delay, seizures. Neuroimaging: comunicating hydrocephalus, macrocephaly	Pancreatic insufficiency.During feve gastrointestinal upset	Retinitis pigmentosa				Splenomegaly
5 [1, 2]	F	Heterozygous: c.668 T.C p.I223T Exon 6Missense	Neonatal	+		SA	Immunodeficiency B-cell	+	Low	Developmental delay, ataxia cerebellar signs	During fever episodes Gastrointestinal upset (abdominal pain, diarrhoea and vomiting)		sensorineural deafness			Cardiomyopathy
6 [1, 2]	M	Compound heterozygous: c.218_219ins22 NA Exon 3 Frameshift c.668 T.C p.I223T Exon 6 Missense	7 m	+		SA	B-cell fluctuated (acute drop during febrile episodes)	+	Low	Developmental delay, seizures, ataxia cerebellar signs	During fever episodes Gastrointestinal upset (abdominal pain, diarrhoea and vomiting)					Splenomegaly, Aminoaciduria + hyperalaninemia
7 [1, 2]	F	Homozygous c.668 T.C p.I223T Exon 6 Missense	3 m	+		SA	B-cell fluctuated	±	low/normal	Developmental delay, seizures, ataxia cerebellar signs. Neuroimaging: cerebral atrophy, abnormal enhancement of external capsule and thalamus	During fever episodes Gastrointestinal upset (abdominal pain, diarrhoea and vomiting)		sensorineural deafness			Splenomegaly, Hypercalciuria Aminoaciduria + hyperalaninemia
8 [1, 2]	F	Compound heterozygous: c.497 T.C p.L166S Exon 5 Missense c.461C.T p.T154I Exon 4 Missense	7 m	±		SA	Immunodeficiency B-cell	±	low/normal	Developmental delay and ataxia cerebella sign. Neuroimaging: delayed white matter myelination	During fever episodes Gastrointestinal upset (abdominal pain, diarrhoea and vomiting)		sensorineural deafness		torticollitis	Nephrocalcinosis, Aminoaciduria + hyperalaninemia
9 [1, 2]	F	Homozygous: c.569G.T p.R190I Exon 5 Missense	7 m	+		SA	Progressive fall in B and T cells	+	Low	Developmental delay	During fever episodes Gastrointestinal upset (abdominal pain, diarrhoea and vomiting)					Cardiomyopathy and Hypertension
10 [1, 2]	M	Compound heterozygous: c.668 T.C p.I223T Exon 6 Missense c.1057-7C.G NA Intron 7 Splicing	7 w	+		SA	Increased naive IgD CD27 B-cells, leaky maturation arrest in B precursors	+	Low	Developmental delay	During fever episodes Gastrointestinal upset (abdominal pain, diarrhoea and vomiting)	Retinitis pigmentosa			Non specific metaboli myopathy	Aminoaciduria + hyperalaninemia, lactici acidosis
11 [1, 2]	M	Compound heterozygous: c.472A.G p.M158V Exon 4 Missense c.977 T.C p.I326T Exon 7 Missense	Neonatal	+		SA	Mild immunodeficiency	±	Low normal	-	During fever episodes Gastrointestinal upset (abdominal pain, diarrhoea and vomiting)					
12 [2]	F	Compound heterozygous: c.668 T.C p.I223T Exon 6 Missense c.1142insATGT p.W381fs Exon 8 Frameshift	Neonatal	+	+	SA	Immunodeficiency B and T CD8 cell. Cytokine profile of blood: high levels of IL-1β, IL-6 and TNFα and mildly raised IL-10	+	Low	Mild developmental delay	During fever episodes Gastrointestinal upset (abdominal pain, diarrhoea and vomiting)					Renal distal tubular acidosis
13 [26]	F	Homozygous: c.C443T: p.A148V Exon4 Missense	3 w	+	+	SA	-	-	-	Developmental delay, Seizures, central apnea, acquired microcephaly. Neuroimaging cortical atrophy	Normal		neurosensory deafness			Renal Fanconi syndrome, nephrocalcinosis. Metabolic acidosis, hyperlactatemia and hyperalaninemia. Muscle, liver and kidney biopsy: increased of enlarged mitochondria
14 [26]	M	Compound heterozygous: c.383A > G p.D128G Exon 4 Missense c.518A > T p.Y173F Exon 5 Missense	3.5 Y	-	-	-	-	-	-	3.5y ataxia, dysarthria, gross motor regression, hypotonia, ptosis, horizontal ophthalmoplegia. At 5 y and 8 m hypotonia, ptosis and horizontal ophthalmoplegia, speech slow and dysarthric Neuroimaging:abnormal signals in brainstem and dentate nucleus						
15 [6]	M	Compound heterozygous c.126_128delAGA Exon 2 Deletion c.1246delA [6] Exon 8 Deletion	1y	+	-	microcytosis with anisocytosis, normal value of hb, poikiocytosis with eliptocytes				No		Retinitis pigmentosa, diffuse outer retinal atrophy with foveal preservation			Arthritis, JIA (first year of life)	
16¥ [6]	M	Compound heterozygous: C.609-26 T > C Intron 5 Splicing c.1246A ins[8], p.S418fs Exon 8 Frameshift	21y	-	-	Microcytosis and anysocytosis				No		Retinitis pigmentosa, mild pallor of optic nerve, macular oedema			-	
17¥ [6]	M	Compound heterozygous: C.609-26 T > C Intron 5 Splicing c.1246A ins[8], p.S418fs Exon 8 Frameshift	18 y			Microcytosis and anysocytosis				No		Retinitis pigmentosa, optic disc pallor, macular oedema			-	
18^ [7]	F	Homozygous: c.295C > T R99W Exon 3 Missense	9 m	+	+	Borderline microcytic hypochromic anaemia	Immunodeficiency B	+	Low	Microcephaly		5 y Bilateral subcapsular cataracts, 10y slight optic disc pallor		sparse hair		GH deficiency
19^ [7]	M	Homozygous: c.295C > T R99W Exon 3 Missense	7 m	+	+	Borderline microcytic hypochromic anaemia	Immunodeficiency B	+	Low	Balance problem, microcephaly		6 y subcpsular cataracts, mild disc pallor		sparse hair		Accessory nipple
20^ [7]	F	Homozygous: c.295C > T R99W Exon 3 Missense	4 m	+	+	Borderline microcytic hypochromic anaemia	Immunodeficiency B	+	Low	Microcephaly		2 y cataracts and sever macular dysfunction	Sensorineural deafness	sparse hair		ERGs undectable, He showed also had identified ahomozygous mutation, c.71G > A (p.Trp24*) in GJB2
21ª [4]	F	Compound heterozygous: c.342 + 5G > T Intron 3 Splicing c.668 T.C p.I223T Exon 6 Missense	2 w	+	+	Sideroblastic anaemia, neutropenia and thrombocytopenia. Bone marrow sideroblasts and abundant megakaryopoiesis with dysplastic nuclear morphology	Immunodeficiency B	+	Low	Developmental delay, episodes of encephalopathies with nistagmus and photophobia. Neuroimaging Progressive cerebellar atrophy, widespread abnormalities	Recurrent diarrhoea, vomiting, Hypertransaminasemia, Pancreatic insufficiency, partial villous atrophy	Retinal dystrophy				Splenomegaly, Metabolic acidosis, hyperlactatemia, electrolyte imbalance
22ª [4]	M	Compound heterozygous: c.342 + 5G > T Intron 3 Splicing c.668 T.C p.I223T Exon 6 Missense	3 w	+	+	Sideroblastic anaemia, neutropenia and thrombocytopenia	B-cell immunodeficiency	+	Low	Central hypotony, seizures Neuroimaging at 10 months: multiple focal lesions int he cerebral hemispheres and cerebellum	Hepatomegaly, exocrine pancreatic insufficiency, diarrhoea	Retinal pigmentation				Splenomegaly
23 [13, 18]	F	Compound heterozygous: c.1057-7C.G NA Intron 7 Splicing c.1213G > A G405R Exon 8 Missense	2 m	+	+	Microcytic anaemia	Immunodeficiency B (low B-cell and memory B-cells). Interferon signature positive (INFa)	+	Low	No				Panniculitis or vulvitis or adenitis during fever episodes. Erythematous skin nodules. Skin Biopsy: lobular and septal neutrophilic panniculitis		
24 ~ [8]	F	Compound heterozygous: c.608 + 1G > T Intron 5 Missense c.668 T.C p.I223T Exon 6 Missense	neonatal	-	-	Severe anaemia, raised WBC, numerous circulating NRBC, widespread extramedullary haematopoiesis with accumulation of myeloid cell and NRBCs in all tissues. Bone marrow: excess of erythroid and myeloid cells with hemosiderin				Intracranial bleeding	Hepatomegaly and jaudinece					Splenomegaly
25 ~ [8]	M	Compound heterozygous: c.608 + 1G > T Intron 5 Missense c.668 T.C p.I223T Exon 6 Missense	Neonatal	-	-	SA, raised WBC, extramedullary haemopoiesis. Bone marrow: sideroblasts	Immunodeficiency B	+	Low	Nothing	meconium ileus requiring ileostomy, gastric perforation				proximal upper limb shortening	Cardiomyopathy: right ventricular hypertrophy with asymmetric septal hypertrophy, patent ductus arteriosus. penoscrotal hypospadias and microphallus
26 [10]	F	Homozygous	3 y	+	NR	Pyropoikilocytosis, hemolytic anaemia	Immunodeficiency B	+	Low	Seizures during fever episod MRI subocortical infarcts and periventricular ischemia (possible disseminated encephalomyelitis)	Recurrent gastrointestinal symptoms during fever		Sensorineural deafness			
27 [11]	F	Compound heterozygous: c.495-498del, p.F167Tfs ∗ 9, Exon 3 Deletion c.1246A > G, p.K416E Exon 8 Missense	3 m	+	+	Anaemia	Immunodeficiency B	+	Low	neurological delay			Normal	Panniculitis		
28 [11]	M	Homozygous: c.C295T, p. R99W Exon3 Missense	11 m	No	NR	Microcytic hypochromic anaemia. No sideroblasts	Progressive B-cell immunodeficiency, low level NK, Low transitional B-cell. Elevated IL6 and INFa and interferon signature	+	Low	Mild developmental delay IQ 65–80	Inflammatory Bowel disease Crohn like disease	Congenital cataract	Sensorineural deafness	Severe dermatitis		Dysmorphic facial featres: mild ptosis short philtrum, thin upper lip, low hairline
29 [12]	M	Compound heterozygous	22 m	+		SA, target cells, nucleated red blood cells and basophilic stippling. Anysopoikilocytosis. Bone marrow: Siderobalst, abnormal megakaryocytes	B-cell immunodeficiency	+	Low	Developmental delay						Cardiomiopathy
30 [12]	M	Compound heterozygous	10 m	+		SA, target cells, nucleated red blood cells and basophilic stippling. Anysopoikilocytosis. Bone marrow: Siderobalst, abnormal megakaryocytes	B-cell immunodeficiency	+	Low	Developmental delay				Brittle hair		
31 [14]	M	Compound heterozygous: c.1057-7C.G NA Intron 7 Splicing c.1092A > T Exon 8 Missense	5 m	+	+	SA, anysopoikylocytosis, target cell, ellyptocytes. Bone marrow: sideroblasts				Developmental delay (predominantly gross motor)	emesis, elevated liver enzyme		Sensorineural deafness	Thinning hair		Gh deficiency
32¨ [3]	F	Homozygous: c.644A > G, p.His215Arg Exon 6 Missense	1 m	+	NR	Chronic hypochromic, microcytic anaemia	Lymphopenia during fever	No	No	vertigo, febrile seizures	Oral ulcers, vomiting, abdominal pain, diarrhoea with fevers Hepatosplenomegaly	Normal	Normal	oral ulcers	Arthritis	Splenomegaly
33¨ [3]	F	Homozygous: c.644A > G, p.His215Arg Exon 6 Missense	2 y	+		SA, acute on chronic anaemia	Low-Normal B-cell. Toxic granulosis independently of fevers, low-normal NK, fluctuanting monocytes	No	Low-Normal	Dizziness/vertigo, opsoclonus (during fever) mild developmental delay	oral ulcers, vomiting, abdominal pain, diarrhoea with fevers	Normal	Normal	Normal	Normal	Splenomegaly, GH deficiency. Mild facial dysmorphism
34 [3]	M	Compound heterozygous: c.488A > T, p.Asp163Val Exon 8 Missense c.668 T.C p.I223T Exon 6 Missense	Neonatal	+		SA. Hemophagocytosis on bone marrow aspirate during flares, high ferritin and elevated LFTs (incomplete criteria for secondary HLH)	B-cell immunodeficiency and low normal NK	+	Low/normal	Developmental delay, hypotonia. Seizures; suspected CNS MAS. Abnormal brain MRI (end stage cerebral damage; hemorrhage, volume loss and leukomalacia)	Pseudoobstruction with ileostomy at birth. Necrotizing enterocolitis 6-week-old. Feeding intolerance/TPN dependence. Elevated liver enzymes during fevers. Hepatosplenomegaly	Optic nerve atrophy	Not tested	Normal	Normal	Splenomegaly
35 [3]	F	Compound heterozygous: c.295C > T R99W Exon 3 Missense c.488A > T, p.Asp163Val Exon 8 Missense	3ws	+	+	SA. Evidence of hemophagocytes on bone marrow aspirate smear	B-cell Immunodeficiency Fluctuating neutropenia, monocytopenia Toxic granulosis independently of fevers. Cytokines: increased Il6, IL18, INF signature, TNF receptors	+	Absent	Developmental delay and speech delay. Mildly wide based gait, intermittent opsoclonus and nystagmus (worse with fevers) Normal brain imaging	Oral mucosa and tongue ulcerations, diarrhoea with fevers; transient pancreatic insufficiency, feeding intolerance, chronic constipation. GI biopsy: acute focal colitis Hepatosplenomegaly, elevated liver enzymes	Early retinal degeneration	Sensorineural deafness	Cellulitis, subcutaneous teneder and nodules, Spars hairs	Elevated muscle enzymes	Splenomegaly, Short stature, Dysmorphic features
36 [3]	F	Compound heterozygous: c.295C > T R99W Exon 3 Missense c.488A > T, p.Asp163Val Exon 8 Missense	6 ws	+		Chronic hypochromic, microcytic anaemia. Bone marrow: rare sideroblasts	B-cell Immunodeficiency and low NK	+	low	Developmental delay, improved Mild deficits (mild ataxia and proximal muscle weakness, mild balance difficulties) Absent reflexes at the ankles	History of oral ulcers and tongue swelling with fevers, feeding intolerance, prolonged diarrhoea. GI biopsy: acute and chronic inflammation in the mucosa of the stomach, terminal ileus, colon. Hepatosplenomegaly	Retinitis pigmentosa, retinal degeneration, optic nerve atrophy, bilateral cataract	Sensorineural deafness	Subcutaneous nodules, erythematous rash, cellulitis	Arthritis and myositis. Mononuclear myofasciitis	Splenomegaly, Small kidney, mild dysmoprhic features
37» [3]	M	Compound heterozygous: c.329C > T, p.Thr110Ile Exon 3 Missense c.383A > G p.D128G Exon 4 Missense	6 y	+		SA, pyropoikilocytosis	B-cell immunodeficiency	+	Low	Mild developmental delay, resolved, ADHD	Mild symptoms with fevers (nausea, vomiting, diarrhoea) Hepatosplenomegaly	retinitis pigmentosa		Normal		Splenomegaly and asthma
38» [3]	F	Compound heterozygous: c.329C > T, p.Thr110Ile Exon 3 Missense c.383A > G p.D128G Exon 4 Missense	4 w	+		SA, pyropoikilocytosis	Combined B and T immunodeficiency	+	Low	Mild developmental delay	Mild symptoms with fevers (nausea, vomiting, diarrhoea) Nodular regenerative hyperplasia and hemosiderosis seen in liver autopsy postmortem	Retinitis pigmentosa		Panniculitis		Asthma
39 [3]	M	Compound heterozygous: c.1245_1246insA, p.Ser418Lysfs*9 Exon 8 frameshift c.1246A > G, p.K416E Exon 8 Missense	2 m	+	+	SA	B-cell immunodeficiency, normal NK	+	Low, normalized at 2 y	Developmental delay	Diarrheoa during fever	Bilateral cataract	Sensorineural deafness	Recurrent swelling of digits		
40 [3]	F	Compound heterozygous: c.668 T.C p.I223T Exon 6 Missense c.1245_1246insA, p.Ser418Lysfs*9 Exon 8 frameshift	3 w	+		SA	Combined B and T immunodeficiency	+	Low	Developmental delay	Protein losing enteropathy. Feeding intolerance/ TPN dependence. Pancreatic insufficiency	Retinitis pigmentosa	Sensorineural deafness	Normal		
41 [15]	M	Homozygous: c.668 T.C p.I223T Exon 6 Missense	8 m	+	+	Pancytopenia and SA				Leigh Encephalopathy (8 m and 4 y), slow degeneration of cognitive, language and motor functions, spasticity, dystonia and focal dyscognitive seizure. MRI Symmetric hemorrhagic lesion in thalamus, brain stem and cerebellum	Recurrent gastrointestinal symptoms during fever, malabsorption	Retinis pigmentosa and optic atrophy	Sensorineural deafness	NR	NR	Leigh syndrome
42 [16]	M	Compound heterozygous: c.565 T > C, p.Ile155Thr Exon 4 Missense c.608 G > A, R203K Exon 5 Missense	3 m	+	+ (IUGR)	SA Bone Marrow: Sideroblast	B and T cell Immunodeficiency (low B naive cell and memory B-cells). At 21 months T cell CD4 + deficit. Low TRECs and KRECs	+	Low	Mild neurodevelopmental delay	Gastrointestinal symptoms during fever					Hypertrophic cardiomyopathy
43 [17]	F	Compound heterozygous: c.608 G > A, R203K Exon 5 Missense c.1246A > G, p.K416E Exon 8 Missense	1 m	+	+	microcytic anaemia	Hypogammaglobulinaemia. Normalization of Ig at 22 months	+		developmental delay and microcephaly	Diarrhoea during fever	Cataract	Sensorineural deafness	Brittle hair,cutaneous lesion during fever episodes	oedema of hands during fever episodes	Splenomegaly, facial dysmorphism, GH deficit and others
44 [17]	F	Compound heterozygous: c.938delT; p.Leu313fs Exon 7 frameshift c.1246A > G, p.K416E Exon 8 Missense	4 m	+	+	Mild anaemia	B-cell immunodeficiency	+	Low	developmental delay and microcephaly	Hepatosplenomegaly	Cataract	Sensorineural deafness	brittle hairs, painful cutaneous lesions	Arthritis	Splenomegaly, facial dysmorphism
45 [5]	M	Homozygous: c.218_219ins22 NA Exon 3 frameshift	0 m	+		SA	B-cell immunodeficiency	+	Low	Psycomothor delay, nistagmus	Vomits and diarrhoea	nystagmus		Wolly hair		Metabolic acidosis, facial dysmorphism
46 [5]	F	Compound heterozygous: c.668 T.C p.I223T Exon 6 Missense c.829G > T; p.Glu277* Exon 7	0 m	+	+	SA	B-cell Immunodeficiency	+	Low	Hypotonia, psychomotor delay, behaviour disorders	Vomits and diarrhoea			Fine and brittle hair	Pectus escavatum	Fish mouth
47 [5]	F	Homozygous: c.977 T.C p.I326T Exon 7 Missense	12 m	No	-	SA	Not present	Not present	Not present	Psychomotor delay, hypotonia, ataxia				trichothiodystrophia, dermal ichthyosis, brittle hair	Ligamentous laxity	Cardiac malformation
48 [18]	F	Homozygous: c.977 T.C p.I326T Exon 7 Missense	Neonatal	+	+ (IUGR)	SA, resolved spontaneously at 4 y	B-cell Immunodeficiency, Nk and TCD8 lymphopenia. Activation type I IFN pathway	+	Very low	Developmental delay	Vomits and diarrhoea during fever episodes					
49 [19]	F	Compound heterozygous: c.668 T.C p.I223T Exon 6 Missense c.1057-7C.G NA Intron 7 Splicing	infancy	+		SA	B-cell Immunodeficiency	+	Low		Diarrheoa during fever	Retinitis pigmentosa	Sensorineural deafness	Lichen sclerosus atrophicus and morphea	Osteoporosis	Hypothyroidism
50 [20]	M	Compound heterozygous: c.295C > T R99W Exon 3 Missense c.1234C > T, p.Arg412X Exon 8 Stop	1 m	+	+	Microcytic anaemia. In the time he develops pancytopenia	B-cell immunodeficiency. Decreased percentage of folliculr T helper. B-cell lineage stopped at pre- B-cell stage	+	Absent							Short stature, abnormal gonadal development, albinism
51 [21]	F	Heterozygous: c.448C > T,p.R150C Exon 4 Missense	3 m	+		Microcytic anaemia. Bone marrow normal	NO	NO	Normal	hemiconvulsion and dysrhythmic				erythema and swelling of both hands, feet, knees And face	Arthritis	
52 [22]	F	Compound heterozygous: c.525delT; p.Leu176X, Exon 5 deletion c.938 T > C; p.Leu313Ser Exon 7 missense	7 m	+	+	Microcytic anaemia. Bone marrow normal	B-cell Immunodeficiency (at 4y normal total B-cell but compromised memory B-cells). Low NK, increased Tgd. Cytokine profile: increased IL2 R, IL6, IL8, TNFa	+	Low	Developmental delay				Cellulitis		
53 [23]	M	Compound heterozygous: c.383A > G p.D128G Exon 4 Missense c.1168G > A Gly390Ser Exon 8 Missense	4 m	+	+	SA	B-cell immunodeficiency,NK fluctuant. Normal interferon signature	+	Very Low	Very mild developmental delay, mild instability, mild speech delay. Brain MRI normal	Vomiting and diarrhoea during episodic fever, hypertransaminasemia, Mouth ultcers	Normal	No	Episodic welling of fingers/cellulitis. Eczema and Brittle hair	Arthritis	Normal
54 [24]	M	Homozygous mutation c.914A > T, p.Asp305Val Exon 7 Missense	6 m	+	-	Microcytic anaema, anosocytosis, poikilocytosis, basophilic stippling on the peripheral blood smear	B-cell immunodeficiency, inverted CD4/CD8	+	Low	None	None	Normal	Normal	Brittle Hairs	None	Hepatosplenomegaly, Hperferritinaemia, mild mitralic insufficiency
55 [25]	F	Compound Heterozygous c.495_498del, p.F167Tfs * 9 Exon 5 Frameshift c.1246A > G, p.K416E Exon 8 Missense	6 m	+	+	SA	B-cell immunodeficiency, Immunoglobulin deficit	+	Low	Developmental delay	None	Cataract	Sensorineural deafness	Neutrophilic dermatosis	None	
56 [27]	F	Compound heterozygous: c.498_501delATTT.Phe167fs,Exon5 Frameshift c.947C > T p.Ala316Val; Exon 7 Missense	2 m	+	+	No	B-cell immunodeficiency with Hypogammaglobulinaemia	+	Low	Developmental delay, white matter lesions	None	Cataract, strabismus, enophthalmo	No	Brittle hairs	hyperlaxity	Mild facial dysmorphism, blue sclerae, oral apthousis
57 [28]	F	Compound heterozygous: c.407 C > G, p.Ala136Lys Exon 4 Missense c.361 G > A; p.Glu121Lys Exon 4 Missense	1 m	+	-	No	B-cell immunodeficiency, hypogammaglobulinaemia, increased double negative lymphocyte	+	Low	None	None	None	No	Erithema nodosum	Arthritis	None
58 [29]	NA	Heterozygous	NA	+	-	NA	Immunoglobulin replacement, recurrent infections			Development dela, motor disturbance	Gastrointestinal bleeding			Skin manifestation

**Table 2:** List of TRNT1 mutation reported in 57 SIFD patients. h = homozigous

Mutations reported to date	Patients ID
c.644A > G, p.H215A Missense	32 (h), 33 (h)
c.1057-7C.G NA Intron 7 Splicing	3, 4, 10, 23, 31, 49
c.1092A > T missense	31
c.1142insATGT p.W381fs Exon 8 Frameshift	12
c.1168G > A Gly390Ser Exon 8 Missense	53
c.1213G > A G405R Exon 8 Missense	23
c.1234C > T, p.Arg412X	50
c.1245_1246insA, p.Ser418Lysfs*9 Exon 8	39, 40
c.1246A > G, p.K416E Exon 8 Missense	27, 39, 43, 44, 55
c.1246A ins[8], p.S418fs Exon 8 Frameshift	16, 17
c.1246delA [6] Exon 8 Deletion	15
c.126_128delAGA Exon 2 Deletion	15
c.218_219ins22 NA Exon 3 Frameshift	6, 45 (h)
c.295C > T R99W Exon 3 Missense	18 (h), 19 (h), 20 (h), 28 (h), 35, 36, 50
c.329C > T, p.Thr110Ile	37, 38
c.342 + 5G > T Intron 3 splice mutation	21, 22
c.361 G > A; p.Glu121Lys	57
c.383A > G p.D128G Exon 4 Missense	14, 37, 38, 53
c.407 C > G, p.Ala136Lys A136K	57
c.443 T: p.A148V Exon4 Missense	13 (h)
c.448C > T, p.R150C Exon 4 Missense	51 (heterozygous)
c.461C.T p.T154I Exon 4 Missense	8
c.472A.G p.M158V Exon 4 Missense	11
c.488A > T, p.Asp163Val Missense	34, 35, 36
c.495-498del, p.F167Tfs ∗ 9, Exon 3 Deletion	27, 55
c.498_501delATTT, p.Phe167fs	56
c.497 T.C p.L166S Exon 5 Missense	8
c.518A > T p.Y173F Exon 5 Missense	14
c.525delT; p.Leu176X, deletion Exon 5	52
c.565 T > C, p.Ile155Thr Exon 4	42
c.569G.T p.R190I Exon 5 Missense	1 (h), 2 (h), 9 (h)
c.608 G > A, R203K Exon 5 Missense	42, 43
c.608 + 1G > T Intron 5	24, 25
C.609-26 T > C Intron 5 Splice	16, 17
c.668 T.C p.I223T Exon 6 Missense	3, 4, 5 (heterozygous), 6, 7 (h), 10, 12, 21, 22, 24, 25, 34, 40, 41 (h), 46, 49
c.829G > T; p.Glu277, E77X	46
c.914A > T, p.Asp305Val, D305V	54 (h)
c.938delT; p.L313fs deletion Exon 7	44
c.938 T > C; p.L313S missense Exon 7	52
c.947C > T p A316V Missense Exon 7	56

Figure 2 is a schematic representation of the TRNT1 gene, with the different reported mutations at different sites. The two most frequent reported mutations were the c.295C > T R99W Exon 3 (Missense) and the c.668 T.C p.I223T Exon 6 (Missense).

### Clinical Aspects

Fifty-eight patients were identified, including 32 females (56.1%), with a median age at onset of symptoms of 4 months of life (range 0–252 m) and a mean of 18.3 months (Standard deviation 49.01). Of these, 12 were Asian, 26 Caucasian, 2 African, 2 Latin-American, while ancestry was not reported for 16. We recorded 12 instance of familial consanguinity and 15 of disease in relatives.

**Fig. 3:**
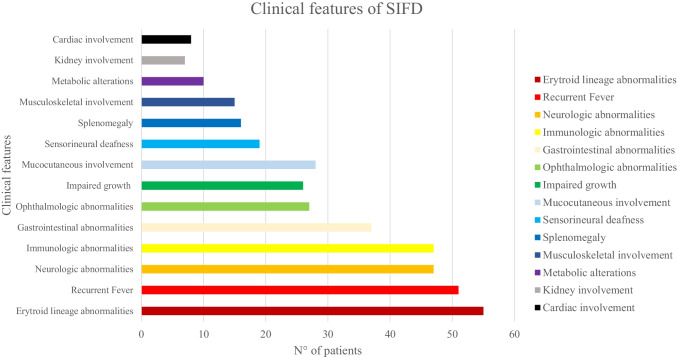
Distribution of main features in 58 patients with SIFD syndrome

The most frequent features were haematologic manifestations such as microcytic anaemia or sideroblastic anaemia (54/58, 93.1%), recurrent fever (52/58, 89.6%), neurologic impairment (48/58, 82.7%), immunologic abnormalities in particular a humoral immunodeficiency (48/58, 82.7%), gastrointestinal signs and symptoms (37/58, 63.7%), eye diseases as cataract and retinitis pigmentosa (27/58, 46.5%), failure to thrive (26/58, 45.8%), mucocutaneous involvement (29/58, 50%), sensorineural deafness (19/58, 32.7%) and others (Fig. 3 and Tables 1, 3 and 4). Only 38/58 (65%) patients showed the complete phenotype characterized by anaemia, recurrent fever, immunodeficiency e neurodevelopmental delay. Nineteen patients died, of whom 3 due to complication of stem cell transplantation.

**Table 3:** Description of treatments performed in each patient and outcomes. *IVIG*, intravenous immunoglobulin; *m*, month; *NR*, not reported; *HSCT*, hematopoietic stem cell transplantation; *AZA*, azathioprine; *MOF*, multi-organ failure

Pts ID and reference	Mutation	Age at onset	Treatments	Outcomes
1* [1, 2, 4]	Homozygous c.569G.T p.R190I Exon 5 Missense	12 m	Red blood cells (RBC) transfusion dependent; Immunoglobulin replacement; Anakinra- later stopped due to allergy	Died at 14 y from sepsis and Multiorgan failure (MOF)
2* [1, 2, 4]	Homozygous c.569G.T p.R190I Exon 5 Missense	2 m	Immunoglobulin replacement; Hematopoietic stem cell transplant (HSCT) performed at 7 y	Alive at 10.5 y after 3 y from HSCT with fever resolution, growth improvement, development progress
3 [1, 2]	Compound heterozygous: c.668 T.C p.I223T Exon 6 Missense c.1057-7C.G NA Intron 7 Splicing	Neonatal	RBC transfusion dependent; Immunoglobulin replacement	Died at 61 months due to MOF
4 [1, 2]	Compound heterozygous: c.668 T.C p.I223T Missense c.1057-7C.G NA Intron 7 Splicing	3 w	RBC transfusion dependent	Died at 56 m due to shock and MOF
5 [1, 2]	Heterozygous: c.668 T.C p.I223T Exon 6Missense	Neonatal	RBC transfusion dependent; Immunoglobulin replacement	Died at 25 months due to cardiac failure secondary to cardiomyopathy
6 [1, 2]	Compound heterozygous: c.218_219ins22 NA Exon 3 Frameshift c.668 T.C p.I223T Exon 6 Missense	7 m	RBC transfusion dependent; Immunoglobulin replacement	Died at 17 months due to MOF
7 [1, 2]	Homozygous c.668 T.C p.I223T Exon 6 Missense	3 m	RBC transfusion dependent; Immunoglobulin replacement	Alive at 19 y
8 [1, 2]	Compound heterozygous: c.497 T.C p.L166S Exon 5 Missense c.461C.T p.T154I Exon 4 Missense	7 m	NR	Alive at 5 y
9 [1, 2]	Homozygous: c.569G.T p.R190I Exon 5 Missense	7 m	RBC transfusion dependent; Immunoglobulin replacement	Died at 28 months cause of myocarditis
10 [1, 2]	Compound heterozygous: c.668 T.C p.I223T Exon 6 Missense c.1057-7C.G NA Intron 7 Splicing	7 w	RBC transfusion dependent; Immunoglobulin replacement up to 9 months when received HSCT	Alive at 4 y (3 y post HSCT). Normal Haemoglobin and IG. Normal growth. Pigmentary retinitis
11 [1, 2]	Compound heterozygous: c.472A.G p.M158V Exon 4 Missense c.977 T.C p.I326T Exon 7 Missense	Neonatal	RBC transfusion dependent	Alive at 45 months
12 [2, P1]	Compound heterozygous: c.668 T.C p.I223T Exon 6 Missense c.1142insATGT p.W381fs Exon 8 Frameshift	Neonatal	RBC transfusion dependent prior to HSCT; Immunoglobulin replacement; HSCT at 13 then at 15 months old	1st HSCT- primary graft rejection 2nd HSCT: Died at 19 months due to multifocal cerebral infarction and progressive renal failure following febrile illness
13 [26]	Homozygous: c.C443T: p.A148V Exon4 Missense	3 w	NR	Died at 21 months because of heart failure during fever
14 [26]	Compound heterozygous: c.383A > G p.D128G Exon 4 Missense c.518A > T p.Y173F Exon 5 Missense	3.5 Y	NR	Alive at 8 years
15 [6]	Compound heterozygous c.126_128delAGA Exon 2 Deletion c.1246delA [6] Exon 8 Deletion	1y	Methotrexate between 4–7 ys	Alive at 19 y
16¥ [6]	Compound heterozygous: C.609-26 T > C Intron 5 Splicing c.1246A ins[8], p.S418fs Exon 8 Frameshift	21y	NR	Alive at 21y
17¥ [6]	Compound heterozygous: C.609-26 T > C Intron 5 Splicing c.1246A ins[8], p.S418fs Exon 8 Frameshift	18 y		Alive at 18y
18^ [7]	Homozygous: c.295C > T R99W Exon 3 Missense	9 m	Immunoglobulin replacement, GH replacement	Alive at 15 y
19^ [7]	Homozygous: c.295C > T R99W Exon 3 Missense	7 m	Immunoglobulin replacement	Alive at 10 y
20^ [7]	Homozygous: c.295C > T R99W Exon 3 Missense	4 m	Immunoglobulin replacement	Alive at 9 y
21ª [4]	Compound heterozygous: c.342 + 5G > T Intron 3 Splicing c.668 T.C p.I223T Exon 6 Missense	2 w	Immunoglobulin replacement, RBC transfusion dependent	Died at 3y and 3 months following a progressive encephalopathy
22ª [4]	Compound heterozygous: c.342 + 5G > T Intron 3 Splicing c.668 T.C p.I223T Exon 6 Missense	3 w	Support therapies	Died at 10 months because of intractable status epilepticus
23 [13, 18]	Compound heterozygous: c.1057-7C.G NA Intron 7 Splicing c.1213G > A G405R Exon 8 Missense	2 m	Immunoglobulin replacement	Alive at 6 year, normal height, no more infections, continued to show 3–4 episodes of fever lasting 1–2 days/month
24 ~ [8]	Compound heterozygous: c.608 + 1G > T Intron 5 Missense c.668 T.C p.I223T Exon 6 Missense	neonatal	Resuscitation treatment	Died at 40 h of age because of MOF and intracranial bleeding (hydrops)
25 ~ [8]	Compound heterozygous: c.608 + 1G > T Intron 5 Missense c.668 T.C p.I223T Exon 6 Missense	Neonatal	RBC Transfusion support HSCT Stem cell transplantation at 5 months	After HSCT developed significant neurological complications and died after 38 days from transplant
26 [10]	Homozygous	3 y	Immunoglobulin replacement 3- to 9 years old, restarted at 10 y	Immunglobulin replacement stopped at 9 years for normal Ig level. Died at 10 for status epilepticus and severe hypoxemia with brain death
27 [11]	Compound heterozygous: c.495-498del, p.F167Tfs ∗ 9, Exon 3 Deletion c.1246A > G, p.K416E Exon 8 Missense	3 m	RBC transfusion	NR
28 [11]	Homozygous: c.C295T, p. R99W Exon3 Missense	11 m	Immunoglobulin replacement systemic corticosteroids for IBD at 20 y AZA for IBD	Alive at 23 y
29 [12]	Compound heterozygous	22 m	NR	NR
30 [12]	Compound heterozygous	10 m	NR	NR
31 [14]	Compound heterozygous: c.1057-7C.G NA Intron 7 Splicing c.1092A > T Exon 8 Missense	5 m	NR	Alive at 21 months
32¨ [3]	Homozygous: c.644A > G, p.His215Arg Exon 6 Missense	1 m	RBC transfusion, systemic corticosteroid. Colchicine	died at 7y because of MOF
33¨ [3]	Homozygous: c.644A > G, p.His215Arg Exon 6 Missense	2 y	RBC transfusion, systemic corticosteroid. Colchicine and Etanercept (8y)	Alive at 11y. Fever suppressed and normalising biomarkers
34 [3]	Compound heterozygous: c.488A > T, p.Asp163Val Exon 8 Missense c.668 T.C p.I223T Exon 6 Missense	Neonatal	Immunoglobulin replacement, Systemic corticosteroid, Cyclosporin A, HSCT	Died a 3 y (92 day post HSCT)
35 [3]	Compound heterozygous: c.295C > T R99W Exon 3 Missense c.488A > T, p.Asp163Val Exon 8 Missense	3ws	RBC transfusion, Immunoglobulin replacement, Anakinra and Etanercept	Alive after 3 y of Etanercept. Fever suppressed and normalising biomarkers
36 [3]	Compound heterozygous: c.295C > T R99W Exon 3 Missense c.488A > T, p.Asp163Val Exon 8 Missense	6 ws	Immunoglobulin replacement, Etanercept switched to Infliximab and AZA	Alive after 12 years of Infliximab. Fever suppressed and normalising biomarkers. Resolution of bowel infiltrate
37» [3]	Compound heterozygous: c.329C > T, p.Thr110Ile Exon 3 Missense c.383A > G p.D128G Exon 4 Missense	6 y	RBC transfusion. Immunoglobulin replacement	alive at 26 years
38» [3]	Compound heterozygous: c.329C > T, p.Thr110Ile Exon 3 Missense c.383A > G p.D128G Exon 4 Missense	4 w	RBC transfusion, splenectomy, Immunoglobulin replacement, oral corticosteroids NSAIDs	Died at 9 years for septic shock
39 [3]	Compound heterozygous: c.1245_1246insA, p.Ser418Lysfs*9 Exon 8 frameshift c.1246A > G, p.K416E Exon 8 Missense	2 m	Immunoglobulin replacement, Anakinra for 3 months discontinued to lack efficacy	Alive, age not specified
40 [3]	Compound heterozygous: c.668 T.C p.I223T Exon 6 Missense c.1245_1246insA, p.Ser418Lysfs*9 Exon 8 frameshift	3 w	RBC transfusions, Immunoglobulin replacement, corticosteroids, Etanercept (started at 21 months)	Alive at 39 months (after 18 months of Etanercept) Fever suppressed and normalising biomarkers
41 [15]	Homozygous: c.668 T.C p.I223T Exon 6 Missense	8 m	RBC transfusion High dose of corticosteroid during Leigh syndrome episode with response	Alive in early adulthood
42 [16]	Compound heterozygous: c.565 T > C, p.Ile155Thr Exon 4 Missense c.608 G > A, R203K Exon 5 Missense	3 m	Immunoglobulin replacement corticosteroid during fever	Died at 26 months for MOF during a sepsi like episode
43 [17]	Compound heterozygous: c.608 G > A, R203K Exon 5 Missense c.1246A > G, p.K416E Exon 8 Missense	1 m	Occasional RBC transfusions, GH replacement Corticosteroid, Anakinra (9 y old) partial response. Etanercept at 11 years still ongoing after 9 years	Alive at 20 y. After Eta starting resolution of fever and associated symptoms
44 [17]	Compound heterozygous: c.938delT; p.Leu313fs Exon 7 frameshift c.1246A > G, p.K416E Exon 8 Missense	4 m	Anakinra for 4 months stopped due to lack of efficacy. Etanercepet started at 2 y still ongoing after 8 y	Alive at 10 years. After Eta starting resolution of fever and associated symptoms
45 [5]	Homozygous: c.218_219ins22 NA Exon 3 frameshift	0 m	RBC transfusions & Immunoglobulin replacement	2 of 3 patients reported by Fouquet died
46 [5]	Compound heterozygous: c.668 T.C p.I223T Exon 6 Missense c.829G > T; p.Glu277* Exon 7	0 m	RBC transfusions & Immunoglobulin replacement	2 of 3 patients reported by Fouquet died
47 [5]	Homozygous: c.977 T.C p.I326T Exon 7 Missense	12 m	RBC transfusions & Immunoglobulin replacement	2 of 3 patients reported by Fouquet died
48 [18]	Homozygous: c.977 T.C p.I326T Exon 7 Missense	Neonatal	RBC transfusions & Immunoglobulin replacement	Alive at 4 years old
49 [19]	Compound heterozygous: c.668 T.C p.I223T Exon 6 Missense c.1057-7C.G NA Intron 7 Splicing	infancy Around 12 m)	Immunoglobulin replacement	Alive at 42 years
50 [20]	Compound heterozygous: c.295C > T R99W Exon 3 Missense c.1234C > T, p.Arg412X Exon 8 Stop	1 m	Immunoglobulin replacement	Alive at 12 years old
51 [21]	Heterozygous: c.448C > T,p.R150C Exon 4 Missense	3 m	Short course of methylprednisolone Etanercept at 5 years of age	Alive at 64 months with an improvement of quality of life after Etanercept starting. The child had no further febrile episodes or seizures
52 [22]	Compound heterozygous: c.525delT; p.Leu176X, Exon 5 deletion c.938 T > C; p.Leu313Ser Exon 7 missense	7 m	Immunoglobulin replacement for 1 year Short term prednisone during fever	Alive at 4 years 10 months, time between attacks increase to 3–4 months
53 [23]	Compound heterozygous: c.383A > G p.D128G Exon 4 Missense c.1168G > A Gly390Ser Exon 8 Missense	4 m	Immunoglobulin replacement since he was 4 months 2 RBC transfusions Anakinra + colchicine for 12 months switched to Etanercept + colchicine	alive at 3 years and 1 month. Since he started Etanercept good control of fever episodes
54 [24]	Homozygous mutation c.914A > T, p.Asp305Val Exon 7 Missense	6 m	Immunoglobulin replacement	Alive at 14 years olds. Stopped Immunoglobulin replacement at 12 years old
55 [25]	Compound Heterozygous c.495_498del, p.F167Tfs * 9 Exon 5 Frameshift c.1246A > G, p.K416E Exon 8 Missense	6 m	RBC transfusions Immunoglobulin replacement Etanerceot	Alive at 4 years with resolution of the main manifestations
56 [27]	Compound heterozygous: c.498_501delATTT.Phe167fs,Exon5 Frameshift c.947C > T p.Ala316Val; Exon 7 Missense	2 m	Immunoglobulin replacement	Alive at 7 years old
57 [28]	Compound heterozygous: c.407 C > G, p.Ala136Lys Exon 4 Missense c.361 G > A; p.Glu121Lys Exon 4 Missense	1 m	Immunoglobulin replacement Corticosteroids Etanercept	Alive
58 [29]	Heterozygous	NA	Immunoglobulin replacement Anakinra Colchicine	Anakinra failure Partial response to Colchicine

**Table 4:** Main features of SIFD syndrome

Genetic features	Mutation IN TRNT1: homozygous mutation or compound heterozygous
Age at onset	In the first months of life Rarely in adulthood
Costitutional symptoms	Recurrent fever: episodes characterized by episodes lasting 5–7 days every 2–4 weeks Failure to thrive
Haematologic features	Sideroblastic anaemia Hypochromic microcytic anaemia Lymphopenia Occasionally neutropenia and thrombocytopenia Blood film: anisopoikilocytosis, hypochromic red cells, elliptocytes and target cell, pencil cells Bone Marrow: Sideroblast or normal. Megakariopoiesis abundant with abnormality
Immunological features	Predominantly Humoral Immunodeficiency Low/absent B-cell Very/low B-cell switched Pan-hypogammaglobulinemia Variable NK and T cell level Increased level of Double Negative lymphocytes Cytokine blood level: Increased IL6, IL18, INFƔ, IP10, MIG Interferon signature: activation of the type I IFN pathway HLH
Neurologic	Developmental delay, hypotonia, Seizures Ataxia, nistagmus Dysarthric- speech delay Horizontal ophthalmoplegia Neuroimaging: cortical atrophy or progressive cerebellar atrophy, abnormal imaging in thalamus or dentate nucleus
Lympho/splee	Splenomegaly Diffuse lymphadenopathies
Gastrointestinal	Recurrent abdominal pain with diarrhoea and vomiting Pancreatic insufficiency Hepatic hypertransaminasemia Partial villous atrophy Chron-like disease Hepatomegaly
Mucocutaneous	Mouth ulcers Eczema Ichthyotic skin Nodular lesions Panniculitis Brittle hair
Renal	Nephrocalcinosis Fanconi syndrome Renal tubulopathy
Hearing	Sensorineural deafness
Ophtalmologic	Retinitis pigmentosa Cataract Optic nerve atrophy
Musculoskeletal	Joint swelling Myopathies
Metabolic abnormalities	Metabolic acidosis, hyperlactatemia, hyperalaninemia
Others:	Cardiomyopathy (typically hypertrophic), High ferritin
Treatment	Blood transfusion Supporting therapy IVIG replacement Supporting therapy Anti-IL1 Anti-TNFα good response. (Possible bridge to HSCT) HSCT resolution of periodic fever and immunodeficiency

The following description is based on the 58 reported cases (Tables 1, 3, 4).

### Haematological Manifestation

Haematologic involvement is the most frequent finding (54/58 patients), usually sideroblastic anaemia (37), while in a minority of cases microcytosis and hypochromasia without prominent ring sideroblasts was reported [1–25]. Peripheral blood smears typically showed hypochromasia, microcytosis, target cell, variable schistocytosis, anisocytosis, elliptocytes, basophilic stippling and nucleate erythrocytes (1–4, 6). Pancytopenia was reported in 4 patients [4, 15, 20] with extramedullary erythropoiesis reported in 2 patients by Barton et al. [8]. Furthermore, in 2 patients, haemophagocytosis was evaluated in the bone marrow, but without fulfilling the HLH-2004 criteria [3, 23].

Bone marrow examination showed in several patients ring sideroblasts that represent more than the 50% of erythroid precursors, erythroid hyperplasia and dyserythropoiesis and in 3 patients, abundant megakaryopoiesis with dysplastic morphology [1–4, 6, 12]. Furthermore, two patients experienced unprovoked recurrent thromboembolic events [3].

### Fever

Recurrent episodes of high spiking fever with upset and elevated inflammatory markers were reported in 52 patients (89.6%) [1–7, 10–29]. Typically, episodes were reported to last for 3–7 days with recurrence every 2–4 weeks or in rare cases every week. Over several years, the interval between attacks had tendency to increase in many cases.

### Immunologic Abnormalities

Immunologic abnormalities were documented in 48/58 (82.75%) patients, all showing an impairment in number of B-cell or circulating Immunoglobulin levels [1–5, 7–25, 27, 28]. Extensive investigation of B-cell subsets highlighted an early defect in B-cell maturation with a block in the pre-B-II stage [1, 20]. Furthermore, Yang et al. described in their patient an impaired number of B memory cell with a reduced number of B switched compared to healthy control [22].

T and Natural killer lymphocytes were fluctuant or decreased in 16 patients [1–3, 9, 13, 16, 18, 20, 22, 23]. Moreover, Wiseman et al. evaluated a progressive decline in T and NK cells seen in several cases of their cohort [1]. High expression of double-negative T cells in the peripheral blood has been reported in 2 patients by Giannaleou et al. [28]. Furthermore Lougaris et al. demonstrated in their patient a severe decrease in T cell receptor excision circles (TRECs) and Kappa-deleting recombination excision circles (KRECs) [16].

Cytokine analysis of blood, performed in some patients during episodes of fever or in the absence of fever, recorded high levels of IL-1β, IL-2R, IL-6, IL-8, IL-18 and TNFα, mildly raised IL-10 and interferon-α induced proteins [3, 9, 18]. The authors of these papers did not specify if the value of these cytokines and interferon-α induced proteins return to the normal value.

### Neurologic Features

Neurological manifestations are reported in 48/58 (82.75%) patients with a widely variable phenotypic spectrum [1–5, 7–12, 14–18, 21–23, 26, 28] (Table 1). The most frequent manifestations are developmental delay or progressive regression in 41 patients, cerebellar symptoms in 15/45 and partial or complex seizures in 15/45 [1–5, 7–12, 14–18, 21, 22, 26]. Brain MRI appearances were widely variable, reporting abnormal results in the 28% cases including cerebral and cerebellar atrophy, hydrocephalus, delayed cortical white matter myelination, lesions in external capsule and thalamus, and hyperintense lesions in the white matter [1–5, 7–12, 14–18, 21–23, 26].

### Gastrointestinal Manifestations

Vomiting, diarrhoea and hepatic disease diseases were described in 38/58 (65%) patients, especially during fever episodes and 5 experienced an exocrine pancreatic insufficiency [1–5, 8–10, 14–19, 23, 29]. Partial villous atrophy or acute and chronic inflammation in bowel mucosa was recorded in 5 patients [3, 4, 9, 19].

### Cutaneous Manifestations

Skin abnormalities were reported in 29/58 SIFD population (49%) mainly brittle hair or ichthyotic skin, panniculitis, erythema nodosum, morfea and aspecific rash [1–5, 7, 9, 11–14, 17–19, 21–25, 27–29]. A biopsy of erythematous nodule lesions showed a lobular and septal neutrophilic panniculitis [13, 18, 25, 28]. Jfri’s adult patient (40 years old) showed lichen sclerosus and atrophicus and morphea [19].

### Ophthalmologic Abnormalities

Ocular involvement was recorded in 27/58 (47.4%) patients, of whom retinitis pigmentosa in 16 and cataract in 11 patients, respectively [1–7, 9, 15, 17, 19, 25, 27].

### Metabolic Abnormalities

Metabolic abnormalities were documented in 10 patients with a wide spectrum of reports including metabolic acidosis, hyperlactacidaemia, hyperalaninaemia, aminoaciduria with increased urinary metabolites of the tricarboxylic acid pathway and in one case Leigh syndrome [1, 2, 4, 5, 15, 26].

Giannelou et al. in their paper described an increased reactive oxygen species (ROS) production in 3 patients’ fibroblasts compare to healthy controls [3]

### Additional Features

Sensorineural hearing loss was described in 20 patients in different age [1–4, 7, 9, 10, 14, 15, 17, 19, 25, 26].

Splenomegaly was reported in 16 patients [1–4, 8, 17, 23, 24], while cardiomyopathy in 7 patients mainly a hypertrophic cardiomyopathy with a case of non-compacted cardiomyopathy [1, 2, 5, 8, 12, 16, 17].

Musculoskeletal involvement was reported in 15 patients of 57 and the most frequent reports are arthritis (8) and myositis (3) and dactylitis [1–3, 5, 6, 8, 17, 19, 21, 23].

Renal disease was reported in 7 patients of these 2 showed Fanconi syndrome, 2 tubulopathy and 4 nephrocalcinosis [1–4, 26].

### Treatment and Outcome

At the time of this systematic review, 19 patients were reported to have died at a median age of 28 months (range 0–168 months): 16 deaths were directly related to the underlying disease course (9 due to multiorgan failure during sepsis-like episodes, 3 to heart failure, 3 to neurologic complications and 1 due to septic shock) and 3 following complications post-stem cell transplantation. Four of the nineteen patients died before five years old. Thirty-three patients remained alive at time of publication (at a median life of 120 months with a range of 21–504 months. Of these 33 patients alive, 16 (48.4%) were older than 10 years old. For 5 patients, clinical outcome was not reported [1–22, 26].

We can distinguish two different therapeutic approaches that have been applied: one supportive, based on blood transfusions, immunoglobulin replacement, antibiotics and electrolyte supplementation; the other with intent to modify the disease course (Table 5). For the latter, hematopoietic cell stem transplantation (HCST) and anti-TNFα drugs have been used with some reports of successful disease modification. HCST was performed in 5 patients as rescue therapy: 3 died due to complications during the first weeks post-transplantation, while 2 remained alive at time of publication with reported complete resolution of episodic fevers, immunodeficiency, haematologic abnormalities and improvement of growth and neurologic features. One developed retinitis pigmentosa during the post-transplant period [1–4, 8].

Anti-TNFα drugs, Etanercept or Infliximab, were used in 10 patients with resolution of fever and associated symptoms, improvement of growth, inflammatory bowel disease and neurologic symptoms [3, 17, 21, 23, 25, 28]. Anakinra, an anti-IL1, was used in 6 patients but discontinued in 5 because of lack of efficacy and in 1 for allergy [1–3, 17, 23]. Colchicine (4/58) and corticosteroids (13/58) were used in attempt to manage febrile episodes, as symptomatic treatment. Anecdotical reports of patients successfully treated with methotrexate (1 patient, due to arthritis) and azathioprine (1 patient, due to inflammatory bowel disease) were also identified [3, 6].

## Discussion

With an expanding landscape of novel, diverse but rare monogenic causes of autoinflammatory syndromes and immunodeficiencies, our first collation of reported cases provides a useful update of the recently described SIFD syndrome [1–4, 13, 18, 20, 30–33]. Our systematic review, summarising the 58 reported cases so far, summarises the spectrum of phenotypic manifestations, treatment approaches and clinical outcomes in this rare syndrome. All reported cases were associated with biallelic mutation in the *TRNT1* gene, with the exception of 3 cases associated with only a discernible heterozygous mutation [1–29].

Mutation in *TRNT1* causes partial loss of function of the TRNT1 RNA polymerase, an essential enzyme necessary for maturation of transfer RNA (t-RNA) in the nucleus and mitochondria, and for respiratory chain function [1–4]. In particular in the mitochondria negatively affect the mature function, with an oxidative phosphorylation defect and decreased enzymatic activities of complexes I, II and IV of the mitochondrial electron transport chain and energy production. This leads to an impaired intracellular stress response, impairment in protein clearance and dysfunctional autophagy. Thus, defective maturation of t-RNA in the mitochondria and cytosol leads to defective protein synthesis, clearance system, dysfunctional autophagy as well as translation of multiple tRNA[1–4, 9]. This mechanism affects not only the iron metabolism in the mitochondria of the erythroblast causing congenital sideroblastic anaemia, but also B-cell lymphocyte maturation, and diverse impacts on several other tissues characterized by high production of protein [3, 9, 19]. The accumulation of cellular stress, secondary to dysregulated protein metabolism, leads to cell destruction and periodic inflammation.

However, based on data of the literature is not possible to say if the metabolic abnormalities, in terms of lactic acidosis or increased of ROS or aminoacidic alterations, are always present or only during fever episodes. Moreover, as a consequence of metabolic abnormalities in several patients, nephrocalcinosis was frequently described in terms of renal tubular acidosis that led to hypercalciuria and electrolytes imbalance. While hyperferritinaemia was reported in several cases, a consequence of the hyperinflammation but also of the iron overload because of diserytropoiesis and blood transfusions (1–3).

While the immunological abnormalities, one of the predominant characteristics of this syndrome, seem to be probably a consequence of defective maturation of B-cell with an increase’s percentage of immature B-cell and decrease production of Immunoglobulins of different classes. TRNT1 enzyme has a crucial role in the protein maturation and degradation, and B-cell differentiation was stopped I at pre-bell steps as demonstrated by Kumaki et al. [20]. Immunoglobulin levels may widely vary reflecting the severity of the enzyme deficiency and B-cell immunodeficiency, and the most affected are the IgG. Moreover, during the episodes of fever, some patients showed a drop of B-cell. However, Wiseman et al. evaluated a progressive decline in the lymphopoiesis in general suggesting a general impact on lymphopoiesis [1].

If we take a carefully look in the different pathogenic variants on TRNT1, it seems that more severe forms are more likely associated with mutations in the catalytic region of the protein, while the milder phenotypes are associated with mutations in the N- or C-terminal or an inheritance in heterozygosis manner. A recent study by Leibovitch showed that while some mutations (e.g., T154I, L166S) impair the protein stability, other mutations (e.g., M158V, R190I and I223T) impact both the CCA-adding catalytic activity as well as protein stability [34]. However, according to the current literature, a clear genotype–phenotype correlation is far to be identified, since patients with the same genotype showed totally different phenotypes with completely different outcomes [8].

SIFD syndrome is heterogenous and patients may not only present with the classic sideroblastic anaemia, B-cell immunodeficiency, fever or developmental delay but can also have a wide variety of other manifestations including retinitis pigmentosa, cataract, inflammatory bowel disease, pseudo-juvenile-arthritis, sensorineural deafness, panniculitis, cardiomyopathy and cutaneous disease configuring a TRNT1 relating disease. Indeed, only the 65% of cases showed the complete phenotype, probably mirroring a different enzymatic residual activity but also a different penetrance of the mutation and the different effect of the environment. A complete correlation between genotype-phenotype, as in other metabolic disease, at the moment is not possible, based on the current knowledge, but future prospective study will help in understanding it. Moreover, sometimes, the typical manifestation may be absent; thus, based on these findings, it would be more appropriate to think this syndrome as a TRNT1-related disorders. Consideration of SIFD in the differential diagnosis could be crucial for prompt and tailored treatment in order to delay its natural history, prevent complications and potentially direct patients to potentially curative allogeneic stem cell transplant. However, the phenotypic spectrum of this novel autoinflammatory disease is still emerging, with some distinctive common features but a diverse set of clinical phenotypes and patterns of system involvement described. Effective recognition, diagnosis and management planning thus mandates increased awareness amongst diverse specialists, including neurologists, haematologists, rheumatologists and immunologists, and close multidisciplinary working for individual diagnosed patients. Furthermore, the increased knowledge about its pathophysiology is opening the route to new possible treatments. Anti-TNFα agents currently represent a real opportunity for these patients while bone marrow transplantation needs to be considered carefully taking into consideration the risk of complications including those correlated to severe lactic acidosis [1, 3, 17]. Maybe a conditioning with TNF-inhibitors will help in the future in these patients even though there are not enough evidence at the moment. Moreover, we need to consider that the treatment may change across the different age of the patients, as the need of red blood cells transfusion seems to decrease based on literature data. As well as the course of the main manifestations of this rare syndrome may be widely variable, based on the different phenotypes.

Two different groups, independently, suggested a fascinating link between mitochondrial disease and interferonopathies, based on the constitutive activation of the type I IFN pathway in patients with biallelic mutations in TRNT1. A potential role of type I IFN in the pathogenesis of SIFD has been therefore suggested [3, 18].

The reactive oxygen species produced by mitochondria are currently emerging as critical factors in signalling pathways regulation, triggering NLRP3 inflammasome activation and type I IFN production [3, 18]. It was also hypothesised that a mutual activation of ROS and type I IFN results in type I IFN-mediated autoinflammatory disease. This might be potential putative mechanisms able to explain the wide range of different autoinflammatory manifestations occurring in SIFD. Therefore, the evidence of a positive interferon signature during the inflammatory episodes might suggest a possible role for JAK-inhibitors. Controlled studies are warranted, but the rarity of this disease hampers this possibility. [2, 3 9, 14, 19, 21].

This systematic review has some limitations. The data extracted from the different papers were not homogenous, due to the different nature of the selected studies, and their different aims. We decided to include single case reports in order to not miss any reporting peculiar phenotype of a such rare disease. We recognize that this may led to a positive selection bias (publication bias), however, the rarity of the disease might also overcome this issue. Notably, we did not include studies when it was not possible to extract information on potentially eligible patients and about their phenotype. The data provided from the different papers are very various and the functional tests, that are crucial to understand the real mechanism of the disease, are not performed in all the patients studied. Considering all these points this systematic review provides a low quality of evidence.

A comprehensive study reporting prospectively the complete natural history of the disease from the onset to the development of the different manifestations and different laboratory abnormalities would be helpful for clinicians. We propose the need for an international, multicentre collaborative registry to collect a comprehensive, standardized dataset to better define the natural history of the disease. The clinical summaries of SIFD syndrome herein provided may be a useful tool to select patients with autoinflammatory syndromes and immunodeficiencies to direct specific testing for TRNT1 gene and therefore starting a more appropriate treatment according to the current knowledge.

## Questions

1. **SIFD may presents with?**
  a. Recurrent Fever
  b. Immunodeficiency
  c. Developmental delay
  d. Anaemia
  e. All of the above *
2. **Which is the most frequent immunodeficiency associated with SIFD?**
  a. T immunodeficiency
  b. Humoral immunodeficiency*
  c. NK deficit
  d. Neutrophil deficit
  e. None
3. **TRNT1 is**
  a. An enzyme essential for the maturation of mRNA
  b. A nucleolar enzyme essential for the maturation of DNA
  c. RNA polymerase, essential enzyme necessary for maturation of transfer RNA (t-RNA) in the nucleus and in the mitochondria and respiratory chain function*
  d. A mitochondrial enzyme
  e. A protein of the respiratory chain function in the mitochondria
4. **Which is the possible therapeutic approach?**
  a. Immunoglobulins, Blood transfusions, corticosteroids
  b. Colchicine and Anti-IL1
  c. IVIG
  d. Based on clinical phenotype with the possibility to use immunoglobulins, blood transfusions, anti-TNF, Hematopoietic stem cell transplantation *
  e. Corticosteroids
5. **Which part of the body can be involved?**
  a. Heart
  b. Eye and ears
  c. Joints
  d. Brain
  e. All of the above*

## Supplementary Information

Below is the link to the electronic supplementary material.

Supplementary file1 (DOCX 13 KB)

Supplementary file2 (DOCX 21 KB)

## Data Availability

All the data available are uploaded in the manuscript or in the supplementary materials.
