# Supplementary material for: Clinical and Therapeutic Aspects of Sideroblastic Anaemia with B-Cell Immunodeficiency, Periodic Fever and Developmental Delay (SIFD) Syndrome: a Systematic Review
Source: J Clin Immunol. 2022 Aug 19;43(1):1–30. doi: 10.1007/s10875-022-01343-0 (PMC9840570; doi:10.1007/s10875-022-01343-0)
Supplement: Supplementary file 1 — Supplementary file1 (DOCX 13 KB) [file 10875_2022_1343_MOESM1_ESM.docx]

Supplementary Materials:

Methods:

The following research keywords in English were used: “TRNT1”, “SIFD”, “sideroblastic anaemia AND TRNT1”, “sideroblastic anaemia AND Immunodeficiency” and “sideroblastic anaemia AND immunodeficiency AND fever”. Language restriction was applied to evaluate only papers written in English. This review included registries, retrospective cohorts, prospective cohort studies, case series and single case reports. Experimental and quasi experimental studies including clinical trials and open label studies reporting data on new cases of SIFD patients have been considered. Animal studies and review articles were excluded.

Title, abstract and full-length text screenings were performed independently by two reviewers (IM and EM) When there was a disagreement, it was resolved through discussion with a third reviewer (GS).

To be eligible for inclusion, the papers were required to a) describe the clinical characteristic of the patients and b) a genetic confirmation of TRNT1 mutation. As exclusion criteria we considered a) the absence of a confirmatory genetic diagnosis, b) not describing the phenotype of patients.

The main outcomes are the clinical and laboratory characteristics of patients with SIFD syndrome.

Two reviewers independently screened the retrieved titles and abstracts and excluded duplicates, those obviously irrelevant, and articles not related to the topic. If the information in the abstracts was insufficient to make a decision, full text was retrieved. Full text of the selected articles was examined to determine whether they satisfied the criteria, and this was confirmed by a second reviewer. The references of all eligible articles including reviews, were manually searched for potentially eligible publications. During consensus meetings, disagreements of selections were resolved. Data were extracted by a single reviewer using a standard form and checked by a second reviewer. The data items extracted included first author, title of study, year of publication, type of study, genetic characteristics, participant demographics characteristics, the clinical phenotype of patients, immunological characteristics, treatment performed, the outcomes and other clinical manifestations showed by each patient. Data were collected and organized using Microsoft Excel and were reported as median, range and percentage.
