# Supplementary material for: Clinical and Therapeutic Aspects of Sideroblastic Anaemia with B-Cell Immunodeficiency, Periodic Fever and Developmental Delay (SIFD) Syndrome: a Systematic Review
Source: J Clin Immunol. 2022 Aug 19;43(1):1–30. doi: 10.1007/s10875-022-01343-0 (PMC9840570; doi:10.1007/s10875-022-01343-0)
Supplement: Supplementary file 2 — Supplementary file2 (DOCX 21 KB) [file 10875_2022_1343_MOESM2_ESM.docx]

Supplemental Table 1: List of all included articles

| Title | *Journal* | Authors | Year | N° of reference |
| --- | --- | --- | --- | --- |
| A novel Syndrome of congenital sideroblastic anemia, B cell immunodeficiency, periodic fevers, and developmental delay (SIFD) | *Blood* | Wiseman et al. | 2013 | 1 |
| Mutation in TNT1 cause congenital sideroblastic anemia with immunodeficiency, fevers, and developmental delay (SIFD) | *Blood* | Chakraborty et al | 2014 | 2 |
| The 3’ addition of CCA to mitochondrial tRNA ser (AGY) is specifically impaired in patients with mutations in the tRNA nucleotidyl transferase TRNT1 | *Human Molecular Genetics* | Sasarman et al | 2015 | 5 |
| Hypomorphic mutations in TRNT1 cause retinitis pigmentosa with erythrocytic microcytosis | *Human Molecular Genetics* | DeLuca et al | 2016 | 6 |
| Expanding the Phenotype of TRNT1-Related Immunodeficiency to Include Childhood Cataract and Inner Retinal Dysfunction | *JAMA ophthalmology* | Hull et al | 2016 | 7 |
| TRNT1 deficiency: clinical, biochemical and molecular genetic features | *Orphanet Journal of Rare Disease* | Wedatilake et al | 2016 | 4 |
| SIFD as a novel cause of severe fetal hydrops and neonatal anaemia with iron loading and marked extramedullary haemopoiesis | *J Clin Pathol* | Barton et al | 2017 | 8 |
| Homozygous N-terminal missense mutation in TRNT1 leads to progressive B-cell immunodeficiency in adulthood | *J Allergy Clin Immunol* | Frans et al | 2017 | 9 |
| Homozygous Trnt1 (Trna Nucleotidyl Transferase 1) Mutation In A Patient With Pyropoikilocytosis And Humoral Immunodeficiency | *Ann Allergy Asthma Immunol* | Eisenberg et al | 2017 | 10 |
| Sideroblastic anemia, immunodeficiency, fever and developmental delay (SIFD) referred as leukemia cutis | *Journal of Clinical Immunology* | Ferraroni et al | 2017 | 11 |
| Bone marrow histopathologic findings in SIFD syndrome: beyond the erythroid lineage. | *Blood* | Mariani et al | 2018 | 12 |
| Inherited Immunodeficiency: A New Association With Early-Onset Childhood Panniculitis | *Pediatrics* | Bader-Meunier et al | 2018 | 13 |
| Congenital sideroblastic anemia, immunodeficiency, periodic fevers, and developmental delay syndrome masquerading as alpha thalassemia variant | *Pediatric Blood and Cancer* | Foster et al | 2018 | 14 |
| Aberrant tRNA processing causes an autoinflammatory syndrome responsive to TNF inhibitors | *Ann Rheum Dis* | Giannelou et al | 2018 | 3 |
| Expanding the phenotype of TRNT1 mutations to include Leigh syndrome | *The Canadian journal of neurological sciences* | Gorodetsky et al | 2018 | 15 |
| Novel biallelic TRNT1 mutations resulting in sideroblastic anemia, combined B and T cell defects, hypogammaglobulinemia, recurrent infections, hypertrophic cardiomyopathy and developmental delay | *Clinical Immunology* | Lougaris et al | 2018 | 16 |
| Etanercept as a successful therapy in autoinflammatory syndrome  related to TRNT1 mutations: a case-based review | *Clinical Rheumatology* | Orlando et al | 2021 | 17 |
| Genotype/phenotype correlations of childhood-onset congenital sideroblastic anaemia in a European cohort | *British Journal of Haematology* | Fouquet et al | 2019 | 18 |
| Mutations in TRNT1 result in a constitutive activation of type I interferon signalling | *Ann Rheum Dis* | Fremond et al | 2019 | 19 |
| Congenital sideroblastic anemia associated with B cell immunodeficiency, periodic fevers, and developmental delay: A case report and review of mucocutaneous features | *SAGE Open Medical Case Reports* | Jfri et al | 2019 | 20 |
| Atypical SIFD with novel TRNT1 mutations: a case study on the pathogenesis of B-cell deficiency | *International Journal of Hematology* | Kumaki et al | 2019 | 21 |
| Periodic fever syndrome with novel TRNT1 variant-possible cause of TRNT1 deficiency or just an incidental finding? | *Pediatric Rheumatology* | Sestan et al | 2019 | 22 |
| Novel biallelic TRNT1 mutations lead to atypical SIFD and multiple immune defects | *Gene & disease* | Yang et al | 2020 | 23 |
| Sideroblastic anaemia, Immunodeficiency, Periodic Fevers, and developmental delay (SIFD) presenting as systemic inflammation with arthritis. | *Rheumatology* | Maccora et al | 2021 | 24 |
| A Novel Homozygous TRNT1 Mutation in a Child With an Early Diagnosis of Common Variable Immunodeficiency Leading to Mild Hypogammaglobulinemia and Hemolytic Anemia | *J Pediatr Hematol Oncol* | Topyildiz E et al | 2021 | 25 |
| Neutrophilic dermatosis: a new skin manifestation and novel pathogenic variant in a rare autoinflammatory disease | *Australas J Dermatol* | Bardou MLD et al | 2020 | 26 |
| Biallelic TRNT1 variants in a child with B cell immunodeficiency, periodic fever and developmental delay without sideroblastic anemia (SIFD variant). | *Immunol Lett* | Rigante D et al | 2020 | 27 |
| Case Report: Expanding Clinical, Immunological and Genetic Findings in Sideroblastic Anemia With Immunodeficiency, Fevers and Development Delay (SIFD) Syndrome | *Front Immunol* | Mendoca LO et al | 2021 | 28 |
